# Left atrial appendage doppler velocity as a predictor of recurrence of atrial fibrillation after transesophageal echocardiogram guided electrical cardioversion

**DOI:** 10.1016/j.ijcha.2023.101268

**Published:** 2023-09-08

**Authors:** Maharshi Raval, Akhil Jain, Rupak Desai, Sajid Siddiq

**Affiliations:** a Internal Medicine, Landmark Medical Center, Woonsocket, RI, USA; b Leukemia, The University of Texas MD Anderson Cancer Center, Houston, TX, USA; c Independent Researcher, Atlanta, GA, USA; d Cardiology, Landmark Medical Center, Woonsocket, RI, USA

**Keywords:** Atrial fibrillation, Electrical cardioversion, Recurrence, Left atrial appendage velocity, Transesophageal echocardiogram

## Abstract

**Background:**

There is a paucity of data on average left atrial appendage emptying velocity (LAAV) measured by doppler during transesophageal echocardiogram (TEE) being able to predict the risk of AF recurrence after electrical cardioversion (ECV).

**Methods:**

Using electronic medical records from a community hospital, retrospective study was conducted after identifying all patients that received TEE-guided ECV. Data pertaining to LAAV, AF recurrence, and variables were obtained and analyzed.

**Results:**

Out of 625 patients receiving TEE-guided ECV, 94 were excluded, and 51 did not convert to sinus rhythm. 480 patients had a successful ECV; out of these, 201 (41.87%) and 243 (50.62%) had a recurrence of atrial fibrillation at the end of 1 month and 3 months, respectively. Low LAAV (<=30 cm/s) was independently associated with an increased risk of AF recurrence at the end of 1 month (aOR 2.37, 95CI 1.5–3.73; p < 0.001) and 3 months (aOR 2.51, 95CI 1.59–3.96; p < 0.001) after TEE-guided ECV.

**Conclusions:**

Low LAAV is associated with a high risk of AF recurrence. Identifying a specific subgroup of individuals at high risk of AF recurrence with the help of pre-ECV LAAV will facilitate the early institution of alternate treatment strategies and the plan for additional therapies.

## 1 Introduction

Atrial fibrillation (AF) is the most common cardiac arrhythmia worldwide, affecting >46.3 million people. [1] AF poses a significant healthcare burden, being a risk factor for myocardial infarction, ischemic stroke, and heart failure, and is associated with high mortality and hospitalization rates. [2] Electrical cardioversion (ECV) is frequently sought as a treatment strategy with over 90% success rate for symptomatic patients that need a rhythm control strategy and is the treatment of choice in patients with a new diagnosis of AF who present with hemodynamic compromise. [3].

Various studies have been conducted to evaluate the risk factors for the recurrence of atrial fibrillation and the female sex, hypertension, coronary artery disease, electrocardiographic lead II P-wave duration of > 135 ms, second or greater episode of AF, left ventricular ejection fraction (LVEF) < 0.50, mitral valve thickening, and left atrial diameter > 4.5 cm have been suggested to be predictors of recurrence. [4], [5] Left atrial appendage (LAA), a projection from the left atrium (LA), has been regarded as one of the major sources of electrical activities that trigger AF and atrial tachycardia. [6], [7] The average left atrial appendage emptying velocity (LAAV) is a recognized marker of LAA function considering contractility, stunning, and fibrosis. [8] Despite LAAV serving as a marker of LAA function and being able to be easily calculated by Doppler during pre-ECV transesophageal echocardiogram (TEE), there is a paucity of data on the role of LAAV in predicting the risk of AF recurrence after ECV. Thus, we performed this study to evaluate whether LAAV is associated with an increased risk of AF recurrence after ECV.

## 2 Methods

### 2.1 Data Overview/Source

Data were retrospectively collected from the electronic medical records (EMR) of a single community teaching hospital and affiliated outpatient clinics. As our study was retrospective without any patient interventions, we were not required to obtain an institutional board review.

### 2.2 Data selection and study population

Using CPT procedure codes (93312 for TEE and 92,960 for ECV), all patients that underwent TEE-guided ECV for the first time from October 1, 2016, to June 30, 2022, were identified. From the procedure details, data was collected for the LAAV and whether the cardioversion was successful. Different operators performed the procedure, however, the measurement criteria for LAA emptying velocity were adhered to. From the outpatient clinic records, data was collected on AF recurrence and baseline variables. Patients were excluded if LAAV was not measured, follow-up data was not available, or patients died before the completion of a study-defined follow-up period of 1 and 3 months.

### 2.3 Baseline variables

Variables such as age, sex, hypertension, diabetes mellitus, obesity, AF duration, difficult rate control, and presence of antiarrhythmic medication were studied (Table 1A). Patients were considered to be on antiarrhythmic medication if they were on medications like amiodarone, sotalol, dofetilide, or flecainide prior to the cardioversion or initiated to facilitate cardioversion, they were maintained on the therapy after cardioversion and had AF recurrence while on the medication. Difficult rate control was defined as the need for a maximum recommended dose of beta-blockers or calcium-channel blockers or more than or equal to half the maximum recommended dose of beta-clockers plus calcium-channel blockers prior to cardioversion.

**Table 1A:** Demographics and comorbidities stratified based on LAAV.

	LAAV >=30 cm/s	LAAV < 30 cm/s	p-value
Mean age (years)	68.55	70.74	0.022
Female sex	35.03	41.35	0.135
Hypertension	79.25	86.08	0.04
Diabetes Mellitus	22.11	26.16	0.27
Obesity	32.31	33.76	0.72
Difficult rate control	26.62	24.58	0.59
Antiarrhythmic meds	32.42	35.32	0.48

Values are noted in percentage unless specified.

LAAV-Left atrial appendage velocity.

### 2.4 Outcomes

The success of the ECV attempt (Table 1B) and amongst the patients with successful ECV, recurrence of AF at 1-month (Table 2A) and 3-month follow-ups (Table 2B) were the outcomes studied.

**Table 1B:** Predictors of successful electrical cardioversion.

	aOR	LCI	UCI	p-value
LAA velocity < 30 cm/s	0.937	0.44	1.992	0.865
Age > 65 years	1.001	0.964	1.04	0.966
Female sex	0.282	0.101	0.788	0.016
Hypertension	0.725	0.252	2.09	0.552
Diabetes mellitus	1.286	0.517	3.197	0.588
Obesity	0.706	0.319	1.559	0.389
A fib > 6 weeks old	0.99	0.968	1.013	0.395
Difficult rate control	1.216	0.502	2.948	0.665
Antiarrhythmic meds	1.202	0.544	2.656	0.65

aOR-Adjusted odds ratio, LAA-Left atrial appendage, UCI-Upper confidence interval limit, LCI-Lower confidence interval limit.

**Table 2A:** Predictors of recurrence of atrial fibrillation at 1 month.

	aOR	LCI	UCI	p-value
LAA velocity < 30 cm/s	2.37	1.5	3.73	<0.001
Age > 65 years	1.017	0.99	1.04	0.128
Female sex	1.283	0.787	2.09	0.318
Hypertension	1.206	0.658	2.21	0.545
Diabetes mellitus	0.819	0.477	1.405	0.468
Obesity	1.22	0.741	2.01	0.434
A fib > 6 weeks old	1.02	1	1.04	0.047
Difficult rate control	1.808	1.09	2.998	0.022
Antiarrhythmic meds	1.019	0.629	1.652	0.939

**Table 2B:** Predictors of recurrence of atrial fibrillation at 3 months.

	aOR	LCI	UCI	p-value
LAA velocity < 30 cm/s	2.51	1.59	3.961	<0.001
Age > 65 years	1.011	0.989	1.034	0.313
Female sex	0.945	0.581	1.539	0.821
Hypertension	1.246	0.668	2.255	0.468
Diabetes mellitus	0.705	0.409	1.215	0.208
Obesity	1.352	0.819	2.232	0.239
A fib > 6 weeks old	1.019	0.998	1.04	0.074
Difficult rate control	1.755	1.049	2.937	0.032
Antiarrhythmic meds	0.982	0.606	1.591	0.94

aOR-Adjusted odds ratio, LAA-Left atrial appendage, UCI-Upper confidence interval limit, LCI-Lower confidence interval limit

### 2.5 Analysis

Stata (version 16) MP edition (StataCorp. 2019. *Stata Statistical Software: Release 16*. College Station, TX: StataCorp LLC) was used for the statistical analysis. In our study, we compared demographics, comorbidities, other variables, and outcomes using Pearson’s chi-square test for categorical variables and the Student *t*-test for continuous variables. In addition, we used logistic multivariate regression analysis to obtain an adjusted odds ratio (aOR) for our outcomes.

## 3 Results

A total of 625 patients that underwent TEE-guided ECV were included. 94 patients were excluded based on the criteria. Out of the 531 patients studied, 51 patients did not convert to normal sinus rhythm after ECV. 480 patients had a successful CV; out of these, 201 (41.87%) had a recurrence of AF at the end of 1 month, and 243 patients (50.62%) had a recurrence of AF at the end of 3 months.

Baseline variables were stratified based on whether LAAV was < 30 cm/s or ≥ 30 cm/s. Patients with LAAV < 30 cm/s had higher mean age (70.74 years vs. 68.55 years, p = 0.022), female sex (41.35% vs. 35.03%, p = 0.135), hypertension (86.08% vs. 79.25%, p = 0.04), diabetes mellitus (26.16 vs. 22.11%, p = 0.27), obesity (33.76% vs. 32.31%, p = 0.72), less difficult rate control (24.58% vs. 26.62%), and higher use of antiarrhythmic medications (35.32% vs. 32.42, p = 0.48).

Performed multivariable regression analysis shows low LAAV (<30 cm/s) is independently associated with an increased risk of AF recurrence at the end of 1 month (aOR 2.37, 95% CI 1.5–3.73, p < 0.001) and 3 months (aOR 2.51, 95% CI 1.59–3.96, p < 0.001) after TEE-guided ECV. Difficult rate control before CV was also associated with an increased risk of AF at 1 month (aOR 1.808, 95% CI 1.09–2.998, p = 0.022) and 3 months (aOR 1.755, 95% CI 1.049–2.937, p = 0.032). Low LAAV was not associated with the success of the cardioversion (aOR 0.937, 95% CI 0.44–1.992, p = 0.865). Amongst the risk factors analyzed, only the female sex was significantly associated with a decreased chance of successful cardioversion (aOR 0.28, 95% CI 0.101–0.788; p = 0.016).

## 4 Discussion

The role of LAAV in predicting the risk of AF recurrence has been studied in patients undergoing catheter ablation, but for patients undergoing ECV, it is an under-researched topic. Few studies conducted had small sample sizes, and the results were discordant. The results of our study show that LAAV is associated with higher odds of AF recurrence at 1 month and 3 months after ECV. However, it is not associated with the success of ECV.

AF recurrence can be attributed to LA remodeling caused by LA enlargement, hypertrophy, and fibrosis as well. [9] With LA fibrosis, the size of the LA increases, and the voltage decreases, both of which are associated with low LAAV and an increase in AF recurrence. [10], [11] Previous studies have shown that the factors that influence the LAA function also influence LAAV, such as AF type, duration, left atrium diameter and volume, the structure of the LAA, and heart rhythm. [12] LAAV represents the LA contractile and reserve function, which can be affected in the early stage of LA remodeling. [13] As impaired LA function can precede LA expansion, particularly in patients with paroxysmal AF, LAAV is a more sensitive predictor for sinus rhythm maintenance and AF recurrence than LA size. [8].

The initial assessment of LAAV’s role in the prediction of the outcome of ECV was done by Tanabe et al. in a study of 56 patients, who noted that mean LAAV was higher in the patients that remained in sinus rhythm at the end of 6 months (25.6 cm/s vs. 15.3 cm/s, p < 0.01). [14] Another study of 186 patients by Antonielli et al. showed that at 1-year follow-up after ECV, patients who remained in sinus rhythm had higher mean LAAV (41.7 cm/s vs. 27.7 cm/s, p < 0.001) compared to patients who had a recurrence of nonvalvular AF. [15] Multivariable logistic regression analysis noted LAAV > 40 cm/s to have an odds ratio of 5.2 with p < 0.001 in predicting preservation of sinus rhythm during 1-year follow-up. The most recent study of 121 patients by Walek et al. also noted that patients who had failed ECV or had AF recurrence at 1 year had lower mean LAAV (33.8 cm/s vs. 47.1 cm/s, p < 0.001). [16] With the results of our study, it appears that low LAAV can reliably predict an increased risk of AF recurrence. Given the sparse data, it is difficult to state the cut-off value below which patients should be considered for additional or alternative treatment strategies. These could be the addition of antiarrhythmic medications or early planning for catheter ablations. The cut-off value used in this study of LAAV < 30 cm/s needs to be validated by further adequately powered randomized studies.

### 4.1 Limitations

Given the retrospective nature of the study, it has some limitations. There could be the presence of confounding factors not taken into account in this study. Additionally, it is a single-center study limiting the generalizability of the results. We did not obtain data about the morphology of the LAA, LVEF, ventricular filling pressures, and valve disorders. Data regarding underlying cardiac pathology, cavity dilations, clinical presentation (compensated/decompensated), or the indication for electrical cardioversion (emergency/elective) was also not obtained. These characteristics related to the cardiac structure or independent of it could contribute to AF recurrence, and as they were not obtained and studied, can confound our results. We used difficult rate control as a variable in our study, but patients on maximum doses of beta-blockade could be on it due to other reasons like optimal heart failure management. Our study does not differentiate between these indications of rate control medications. While considering the duration of AF, we could not obtain information on the type of fibrillation (persistent, long-standing persistent, or first episode). Despite the limitations, we provide comprehensive data from the largest sample size on the topic.

## 5 Conclusions

AF is a highly prevalent condition, with ECV often used as a treatment strategy for rhythm control. There is a risk of recurrence of AF after ECV, and early identification of the patients at risk could help identify appropriate candidates for ECV and be given consideration for additional and/or alternative therapies. Simple measurement of LAAV using Doppler during pre-ECV TEE can help risk stratify patients. Patients with low LAAV < 30 cm/s may need to be considered for early institution of antiarrhythmic medications and planning for catheter ablations. Robust evidence to support this is lacking and needs to be studied further with adequately powered randomized studies. The role of reduced LVEF or the presence of valve disorders that could contribute to AF recurrence also needs to be taken into account.

## Declaration of Competing Interest

The authors declare that they have no known competing financial interests or personal relationships that could have appeared to influence the work reported in this paper.
